# Soy product consumption and the risk of major depressive disorder in older adults: Evidence from a cohort study

**DOI:** 10.3389/fpsyt.2022.888667

**Published:** 2022-09-02

**Authors:** Tao Zhang, Guojun Jiang, Fudong Li, Xue Gu, Yujia Zhai, Le Xu, Mengna Wu, Hongwei Shen, Junfen Lin

**Affiliations:** ^1^Department of Public Health Surveillance and Advisory, Zhejiang Provincial Center for Disease Control and Prevention, Hangzhou, China; ^2^Nanxun Center for Disease Control and Prevention, Huzhou, China

**Keywords:** soy product, depression, elderly, mental health, China

## Abstract

**Background:**

To explore the association between soy product consumption and the risk of depression in the community.

**Methods:**

In 2014, a total of 10,901 older people were recruited from Zhejiang province, China, and completed food frequency interviews. Participants were followed up over the next 6 years, and depression was assessed at each visit. Finally, 6,253 participants were included in the present study. Mixed effects models were performed to analyze the association by multivariate adjustments for potential confounders.

**Results:**

Over four-fifths of the eligible participants took soy food at least one day per week. The mixed effects model has shown the adjusted odds ratios (95% CI) of high-frequency consumers (4–7 days per week) were 0.46 (0.39–0.54) for depression with a cut-off score of 5, compared with non-consumers.

**Conclusions:**

More frequent soy product consumption was associated with a lower risk of depression.

## Introduction

Major depressive disorder (MDD), also depression, has emerged as the world's second most common disorder after cardiovascular disease, affecting more than 264 million people worldwide (1). Depression in older adults is prevalent in community living settings (2, 3), and increases the risk of mortality (4, 5) and negatively influences the quality of life (6). Therefore, it is important to identify modifiable risk factors for depression in older people for early prevention.

Modifiable factors, such as physical exercise, comorbidity (such as diabetes and arthritis), and dietary factors, play crucial roles in the development of depression, as do non-modifiable factors such as sex (7–10). Previous epidemiologic studies have investigated the relationship between soy food intake and depression although many did so only as part of an overall dietary pattern (11). A cross-sectional study from China involving 11,473 participants aged ≥35 years found that weekly consumption of beans or bean products was negatively associated with depressive symptoms (12). A cohort study from Taiwan indicated no significant associations between legume intake and risk of depression after 4 years of follow-up among 1,609 participants aged ≥35 years (13). The remaining studies included soy intake as part of an overall dietary pattern, and the results were inconclusive (14–17).

Soy products are a prominent source of isoflavones, and epidemiological evidence suggests a relationship between isoflavones and depression (11). As a traditional food, various soy products, such as tofu, soy milk, and tofu sheets, are consumed frequently in Chinese families. We hypothesized that soy product consumption was independently associated with depression. Most of the previous studies were limited in sample sizes or design (12, 13), were too low-powered to obtain effect estimates and were unable to obtain robust results through long-term surveillance. In this study, we aimed to examine the associations of soy product consumption with depression in a community-dwelling older adults prospective cohort study.

## Methods

### Study design and population

Data were obtained from a public health surveillance project aimed at exploring health problems among elderly people in Zhejiang. The design of the project has been fully described before (18, 19). Briefly, seven counties were selected from 90 in Zhejiang province, China in 2014. The counties were chosen according to local disease patterns, exposure to certain risk factors, population stability, quality of death and disease registries, local commitment, and the capacity of staff. In each county, one town was selected, and then each town recruited randomly no fewer than 1,500 permanent residents aged 60 years and older. Face-to-face interviews were completed by well-trained interviewers with a questionnaire that included sociodemographic information, food frequency, work experience, cognition data, and current medical history. A total of 10,901 older people took part in the baseline survey in 2014. In 2015 and 2016, 9,989 cohort participants were re-interviewed using the same questionnaires as those at baseline after excluding death and lost to follow-up. In 2019 and 2020, 6,253 were re-interviewed using the same questionnaires as those at baseline after excluding death and lost to follow-up.

The study was conducted in accordance with the Declaration of Helsinki, and approved by the Ethics Committee of the Zhejiang Provincial Center for Disease Control and Prevention (No. 2021-034-01). Informed consent was obtained from each subject involved in the study.

### Depression assessment

MDD was assessed by the Patient Health Questionnaire-9 (PHQ-9). The frequency of nine depressive-related symptoms in the past 2 weeks was collected for each participant, with higher frequency assigned a higher score. The total score ranges from 0 to 27, with a higher score indicating a more severe level. In this study, we adopted six different cut-off scores for detecting MDD (20, 21).

### Soy products consumption assessment

At baseline, the participants were interviewed about the average frequency of soy product intake per week during the previous year. The frequency ranged from 0 to 7 days per week, and the subjects eating soy products for <1 day per week, at least 1 day per week (1–7 days per week), and at least 4 days per week (4–7 days per week) were recorded as non-consumers, regular consumers, and high-frequency consumers, respectively.

### Statistical analysis

Mixed effects logistic regression model was conducted to estimate the odds ratios (ORs) and 95% CI for the association between soy product consumption and depression during follow-up. Model 1 was a crude model without any covariate, model 2 was adjusted for age (continuous), sex (female or male), and BMI (continuous), and model 3 was further adjusted for education (illiteracy, primary school, or middle school or above), marital status (married or others), family economic status (self-reported: rich, median, or poor), physical exercise (yes or no), smoking status (current smoker, ex-smoker, or never), drinking status (drinker, ex-drinker, or never), hypertension (self-reported: yes or no), diabetes (self-reported: yes or no), arthritis (self-reported: yes or no), the Activities of Daily Living Scale (ADL) scores (continuous), fresh vegetables (≥1 day per week or <1 day per week) and fish/shrimp consumption (≥1 day per week or <1 day per week), and dietary supplements (self-reported: yes or no) based on model 2. The covariates were chosen according to the findings in the literature (12, 13). The midpoint value was assigned to each frequency level and treated as a continuous variable in the model to test the linear trend.

We performed stratified analyses according to prespecified baseline groups: age, sex, BMI, education, marital status, family economic status, physical exercise, smoking status, drinking status, hypertension, diabetes, arthritis, ADL scores, fresh vegetable consumption, fish/shrimp consumption, and dietary supplements. We examined the significance of interaction by the interaction term (the stratifying variable × soy product consumption). All statistical analyses were performed with SAS version 9.2. Two-tailed *P* < 0.05 was considered statistical significance.

## Results

Among the eligible 6,253 participants, the mean age was 68.2 years; 51.56% were women. A total of 5,135 participants (82.1%) consumed soy products at least 1 day per week, and 4,363 of them consumed at least 100 g per day. Table 1 shows the baseline characteristics of the participants by category of soy product consumption. Compared with regular consumers (those who consumed soy products ≥1 day/week), non-consumers were more likely to have a higher proportion of illiteracy and low-income families, to have hypertension, diabetes, and arthritis, to do physical exercise, and to take dietary supplements.

**Table 1:** Baseline characteristics by category of soy products consumption among 6,253 participants.

**Characteristics**				
	**<1 day/week**	**1-3 days/week**	**4-7 days/week**	**Overall**
*n*	1,118	3,420	1,715	6,253
Consumption amount (×50g/day)*	0 (0)	2 (1)	2 (1)	2 (2)
Age (years)	68.2 ± 6.4	67.6 ± 6.2	67.5 ± 6.3	67.6 ± 6.3
Women (%)	54.83	51.26	50.03	51.56
Education (%)				
Illiteracy	49.24	41.96	44.13	43.86
Primary school	42.79	47.75	44.89	46.08
Middle school or above	7.97	10.29	10.97	10.06
Married (%)	76.03	80.85	82.57	80.46
Family economic status (%)				
Rich	8.94	9.24	12.13	9.98
Median	78.00	81.52	81.57	80.91
Poor	13.06	9.24	6.30	9.12
Physical exercise (%)	23.97	20.82	15.80	20.01
Smoking				
Current smoker	72.09	68.86	69.68	69.66
Ex-smoker	18.43	20.56	22.27	20.65
Never	9.48	10.58	8.05	9.69
Drinking (%)				
Drinker	24.06	29.42	26.01	27.52
Ex-drinker	9.03	8.27	5.71	7.76
Never	66.64	62.31	68.28	64.72
BMI (kg/m^2^)	23.9 ± 3.2	23.6 ± 3.1	23.4 ± 3.2	23.6 ± 3.2
Hypertension (%)	49.28	43.25	39.48	43.29
Diabetes (%)	12.61	8.33	8.05	9.02
Arthritis (%)	6.98	3.95	4.26	4.57
ADL score*	0 (0)	0 (0)	0 (0)	0 (0)
Fresh vegetable consumption (≥ 1 day per week)	1,101 (98.5)	3,410 (99.7)	1,710 (99.7)	6,221 (99.5)
Fish/shrimp consumption (≥ 1 day per week)	782 (70.0)	2,491 (72.8)	1,133 (66.1)	4,406 (70.5)
Dietary supplements (%)	13.51	12.81	11.78	12.65

* Median, interquartile.

From 2014 to 2020, 1,478 of 6,253 participants had depression (23.6%) on at least one visit with a cut-off score of 5, and 458 had depression (7.3%) on at least two visits. When the cut-off score varies from 6 to 10, the number of participants with depression on at least one visit was 1,188 (19.0%), 931 (14.9%), 747 (11.9%), 578 (9.2%), 408 (6.5%), respectively, and the number of participants with depression on at least two visits was 324 (5.2%), 250 (4.0%), 186 (3.0%), 144 (2.3%), 102 (1.6%).

After multivariate adjustment, inverse associations were found to be significant between soy product consumption and MDD (Table 2). As compared with non-consumers, the adjusted ORs (95%CI) of high-frequency consumers were 0.46 (0.39–0.54) for MDD with a cut-off score of 5 (P for linear trend <0.001). Similar associations were observed for MDD with a cut-off score of 6, 7, 8, 9, and 10, with the ORs (high frequency consumers vs. non-consumers) being 0.40 (0.33-0.49), 0.41 (0.33-0.50), 0.43 (0.34-0.54), 0.42 (0.33-0.55), and 0.45 (0.33-0.61), respectively (Table 2).

**Table 2:** Adjusted association between soy products consumption and depression.

	**Soy products consumption**			
	**<1 day/week (*n* = 1,118)**	**1–3 days/week (*n* = 3,420)**	**4–7 days/week (*n* = 1,715)**	***P* for trend**
MDD (PHQ-9≥5)				
Model 1*	ref.	0.54 (0.47–0.62)	0.40 (0.33–0.47)	<0.001
Model 2^†^	ref.	0.56 (0.49–0.64)	0.41 (0.35–0.49)	<0.001
Model 3^‡^	ref.	0.60 (0.53–0.69)	0.46 (0.39–0.54)	<0.001
MDD (PHQ-9≥6)				
Model 1*	ref.	0.49 (0.42–0.57)	0.35 (0.29–0.42)	<0.001
Model 2^†^	ref.	0.50 (0.43–0.58)	0.36 (0.30–0.43)	<0.001
Model 3^‡^	ref.	0.55 (0.47–0.64)	0.40 (0.33–0.49)	<0.001
MDD (PHQ-9≥7)				
Model 1*	ref.	0.46 (0.39–0.54)	0.35 (0.28–0.43)	<0.001
Model 2^†^	ref.	0.47 (0.40–0.55)	0.36 (0.29–0.44)	<0.001
Model 3^‡^	ref.	0.51 (0.44–0.61)	0.41 (0.33–0.50)	<0.001
MDD (PHQ-9≥8)				
Model 1*	ref.	0.45 (0.37–0.53)	0.36 (0.29–0.45)	<0.001
Model 2^†^	ref.	0.46 (0.38–0.55)	0.37 (0.30–0.47)	<0.001
Model 3^‡^	ref.	0.51 (0.42–0.61)	0.43 (0.34–0.54)	<0.001
MDD (PHQ-9≥9)				
Model 1*	ref.	0.43 (0.35–0.52)	0.35 (0.27–0.46)	<0.001
Model 2^†^	ref.	0.44 (0.36–0.53)	0.36 (0.28–0.47)	<0.001
Model 3^‡^	ref.	0.49 (0.40–0.60)	0.42 (0.33–0.55)	<0.001
MDD (PHQ-9≥10)				
Model 1*	ref.	0.42 (0.33–0.53)	0.36 (0.27–0.48)	<0.001
Model 2^†^	ref.	0.43 (0.34–0.55)	0.37 (0.27–0.50)	<0.001
Model 3^‡^	ref.	0.50 (0.39–0.63)	0.45 (0.33–0.61)	<0.001

MDD, major depressive disorder.

* Unadjusted model.

† Adjusted for age, sex, and BMI.

‡ Adjusted for age, sex, BMI, education, marital status, family economic status, physical exercise, smoking and drinking status, hypertension, diabetes, arthritis, ADL scores, dietary supplements, fresh vegetable consumption, and fish/shrimp consumption.

**Figure 1:**
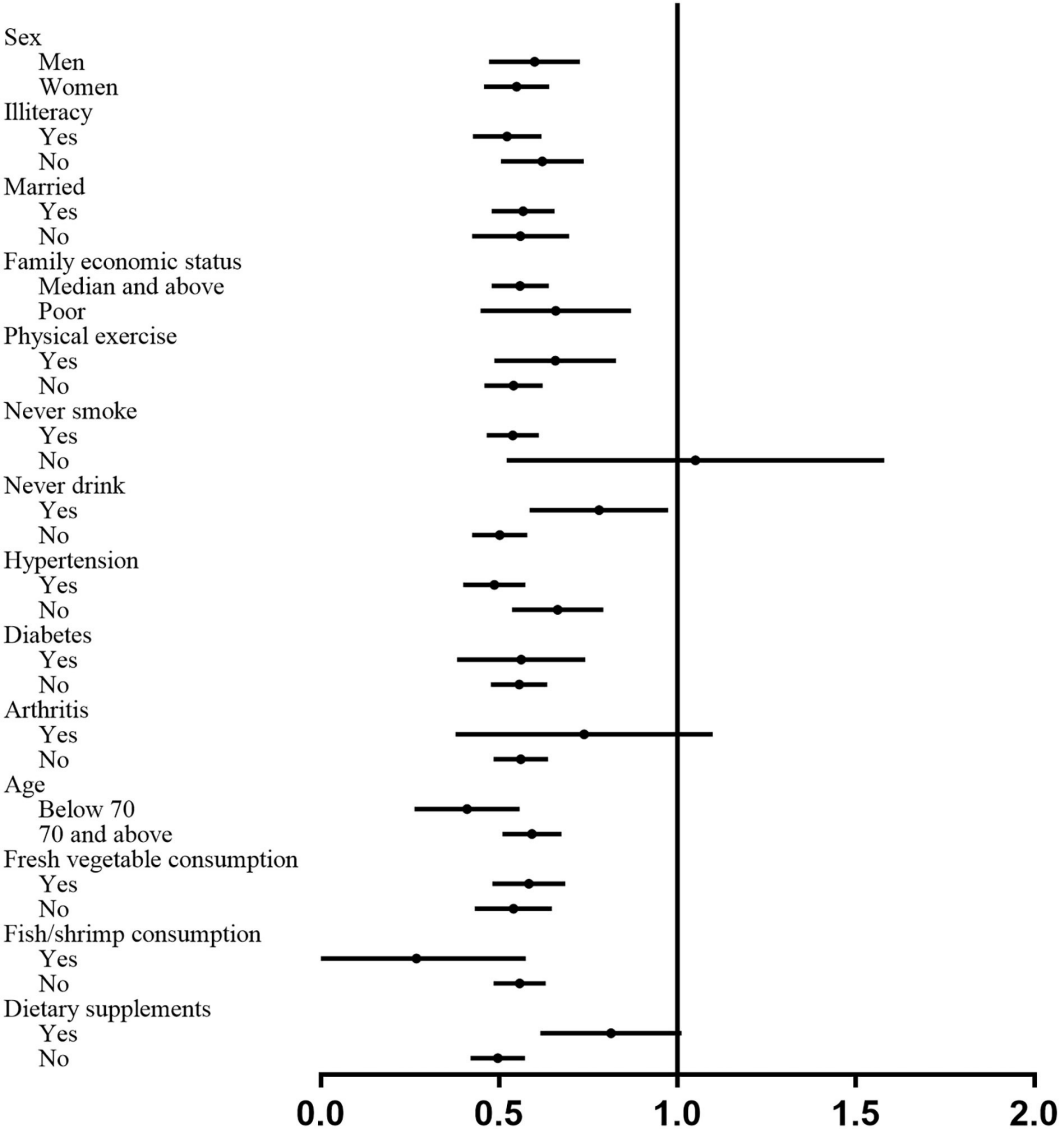
Subgroup analyses of associations between soy product consumption and risk of the major depressive disorder according to potential baseline confounders.

We conducted stratified analyses to examine whether the associations between soy product consumption and MDD were modified by baseline characteristics. No significant interactions have been found across the stratum for age, sex, education, marital status, family economic status, hypertension, diabetes, arthritis, fresh vegetables, and fish/shrimp consumption (Figure 1). Significant differences across the stratum were found, with stronger associations among smokers (P for interaction 0.044), never drinkers (P for interaction 0.007), and subjects with hypertension (P for interaction 0.015). We did not observe a linear trend relationship between weekly soy consumption and depression among the regular consumers, and the OR was 1.00 (95% CI, 0.99–1.01). We performed subgroup analyses among the regular consumers, and the results showed that each 50 g soy product consumption increment per week was associated with an 8% lower risk of MDD (OR 0.92, 95% CI 0.88–0.97) with ≤ 300 g per week, and the dose-effect association was insignificant among the participants eating soy products over 300 g per week (OR 1.00, 95% CI 0.99–1.01).

## Discussion

In this prospective cohort study, we found soy product consumption was associated with MDD, independent of potential confounders. Notably, high-frequency consumers were associated with a 48% lower risk of MDD.

The cut-off score of PHQ-9 is used to differentiate between a subject with or without MDD, and the same cut-off score might not be appropriate in all settings (22). We explored the association between soy and MDD with several different cut-off values according to previous literature (21–23), and the results showed the association was robust.

Results from our study have been consistent with previous studies. A Chinese cross-sectional study reported an inverse association between soy products consumption and depression in rural older people, and individuals consuming soybeans ≥2 times/week had a lower possibility to have depressive symptoms than those rarely consuming (OR (95% CI): 0.36 (0.15, 0.87) for 2–3 times/week and OR (95% CI):0.50 (0.34, 0.74) for ≥4 times/week) (24). Several studies have shown that a dietary pattern rich in soybeans is associated with a low risk of depression (25, 26).

Many Asian soy products are minimally processed, and rich in isoflavones (27). In western countries, intake recommendations seldom exist for soy foods. A study from Franch showed that vegetarian diets were associated with depressive symptoms mostly in participants with low legume intake, and legumes exclusion diet was associated with a 22% increase in risk for depressive symptoms (28). Two recent systemic reviews demonstrated that a vegetarian diet significantly increased depression risk (29, 30). However, both of the studies failed to report the association between soy product consumption and depression. In our study, regular soy product consumption was associated with a lower risk of depression, and the association was independent of fresh vegetables, fish, shrimp consumption, and other potential confounders.

Subgroup analyses showed that the association between soy and depression remained in most subgroups except cigarette smoking. No significant association between soy and depression was found in smokers. A systematic literature review showed that smoking exposure was associated with later depression. It is possible, therefore, that the association is counteracted by cigarette smoking. The insignificant associations observed among the participants with arthritis and dietary supplement intake might be explained in this same way. Fish consumption is commonly recognized as the primary source of methylmercury exposure, and a Korean study suggested that higher blood mercury was positively associated with the risk of depression (31). However, a meta-analysis study reported that high-fish consumption could reduce the risk of depression based on the data from the observational studies, and the findings remained significant in the cohort studies (32). By contrast, several cohort studies did not support the protective role of higher fish intake against mental health (33–35). The SUN cohort study even observed a positive association between fish consumption and the risk of mental disorders (36). Bloch et al. (37) analyzed 13 randomized, placebo-controlled trials, and found no significant benefit of omega-3 fatty acids treatment in MDD. In the present study, the protective effect of soy on depression in regular fish/shrimp consumers tended to be greater than that in non-consumers, but the difference was not statistically significant.

The emerging evidence suggested that isoflavones may function as antidepressants, although the results were inconclusive. An Italian study that was evaluating mood effects found that postmenopausal women taking 54 mg/day of genistein showed a decline in depressive symptoms, whereas no change occurred in the placebo group (38). Also, a Japanese study involving peri- and postmenopausal women found that a very moderate dose (25 mg/day) of isoflavones consumed in aglycone form reduced depressive symptoms (39). In contrast to the benefit of this dose, this eight-week trial found that a very low dose of isoflavones (12.5 mg/day) lacked efficacy. Chedraui et al. found no significant benefits of soy isoflavones treatment on depressive symptoms in climacteric women (40).

Animal experiments suggested the underlying mechanisms of isoflavones. Soy isoflavones might affect monoamine neurotransmitters by reshaping the structure of the gut microbiota, thereby alleviating depression-like behavior (41). S-equol, a major metabolite of dietary soy isoflavones, significantly alleviated the depressive-like behavior in mice (42).

The strengths of our study include a prospective cohort design and the adjustment for a considerable number of potential risk factors for depression. However, this study has some limitations. There is no evaluation of the circulating levels of isoflavones and of the equol-competence that could have better clarified the mechanisms associated with the phenomenon. Soy product consumption was obtained *via* a non-validated qualitative food frequency questionnaire, but the consumption frequency was similar to that reported by the China Kadoorie Biobank (CKB) study in Zhejiang (1–3 days per week, 64.1%, ≥4 days per week, 23.0%, <1 day per week, 12.9%) (43). Depression was assessed by a questionnaire (PHQ-9) rather than the gold standard structured clinical interview diagnostic in our study. However, the PHQ-9 is a reliable and validated instrument, extensively used to assess depression (44–46), and the nine items of PHQ-9 are consistent with the 9 diagnostic symptoms for major depressive disorder in the DSM-5. This study was carried out on an elderly and geographically limited population, and caution must be taken in transferring the results to other populations.

## Conclusions

In brief, the present study found that soy product consumption was associated with a lower risk of depression. Our findings suggest that regular soy food intake may have a beneficial effect on mental health in older adults.

## Data availability statement

Publicly available datasets were analyzed in this study. Due to containing sensitive information, data are available from the Ethics Committee of Zhejiang Provincial Center for Disease Control and Prevention (contact via Zhengting Wang, ztwang@cdc.zj.cn) for researchers who meet the criteria for accessing confidential data.

## Ethics statement

The studies involving human participants were reviewed and approved by the Ethics Committee of Zhejiang Provincial Center for Disease Control and Prevention. The patients/participants provided their written informed consent to participate in this study.

## Author contributions

Conceptualization: TZ and JL. Methodology: FL. Software, formal analysis, and writing original draft preparation: TZ. Validation: FL, GJ, and LX. Investigation: HS, GJ, and XG. Resources and project administration: JL and GJ. Data curation: XG and YZ. Writing review and editing and supervision: JL. Visualization: MW. Funding acquisition: TZ and FL. All authors have read and agreed to the published version of the manuscript.

## Funding

This research was funded by the Zhejiang Provincial Public Welfare Technology Application Research Project of China (LGF21H260002), the Natural Science Foundation of Zhejiang Province (Q19H260001), and the Medical Health Science and Technology Project of Zhejiang Provincial Health Commission (2021KY619).

## Conflict of interest

The authors declare that the research was conducted in the absence of any commercial or financial relationships that could be construed as a potential conflict of interest.

## Publisher's note

All claims expressed in this article are solely those of the authors and do not necessarily represent those of their affiliated organizations, or those of the publisher, the editors and the reviewers. Any product that may be evaluated in this article, or claim that may be made by its manufacturer, is not guaranteed or endorsed by the publisher.
